# P-2317. Risk Factors Associated with post-Hematopoietic Stem Cell Transplant Mortality: Two-Year Cohort in a Public Hospital in Perú

**DOI:** 10.1093/ofid/ofae631.2469

**Published:** 2025-01-29

**Authors:** Mario Jeisson Agramonte, Karol Moscol-Chavez, Lourdes Aranda-Gomero, Alfredo Wong-Chang, Stalin Vilcarromero, Cesar Copaja-Corzo

**Affiliations:** Hospital Edgardo Rebagliati Martins, Lima, Lima, Peru; Hospital Edgardo Rebagliati Martins, Lima, Lima, Peru; Hospital Edgardo Rebagliati Martins, Lima, Lima, Peru; Hospital Edgardo Rebagliati Martins, Lima, Lima, Peru; Hospital Nacional Edgardo Rebagliati Martins. ESSALUD, LIMA, Lima, Peru; Unidad de investigación para la generación y síntesis de evidencias en salud, Universidad San Ignacio de Loyola, Lima, Lima, Peru

## Abstract

**Background:**

In Latin America, Hematopoietic Stem Cell Transplants have experienced significant growth in recent years, both in terms of quantity and quality. Specialized hospitals and high-level medical centers have been established in several countries, including Perú, to carry out these procedures.

The availability of compatible donors, the experience of the medical team, the condition of the recipient, the stage of the disease, the resources available for monitoring and surveillance of transplant complications are some of the factors involved in the success of the transplant.

This study aims to describe the characteristics and factors associated with mortality in hematopoietic transplant recipients in patients post hematopoietic stem cell transplant attended at a hospital in Peru

sociodemographic characteristics

**Figure:**
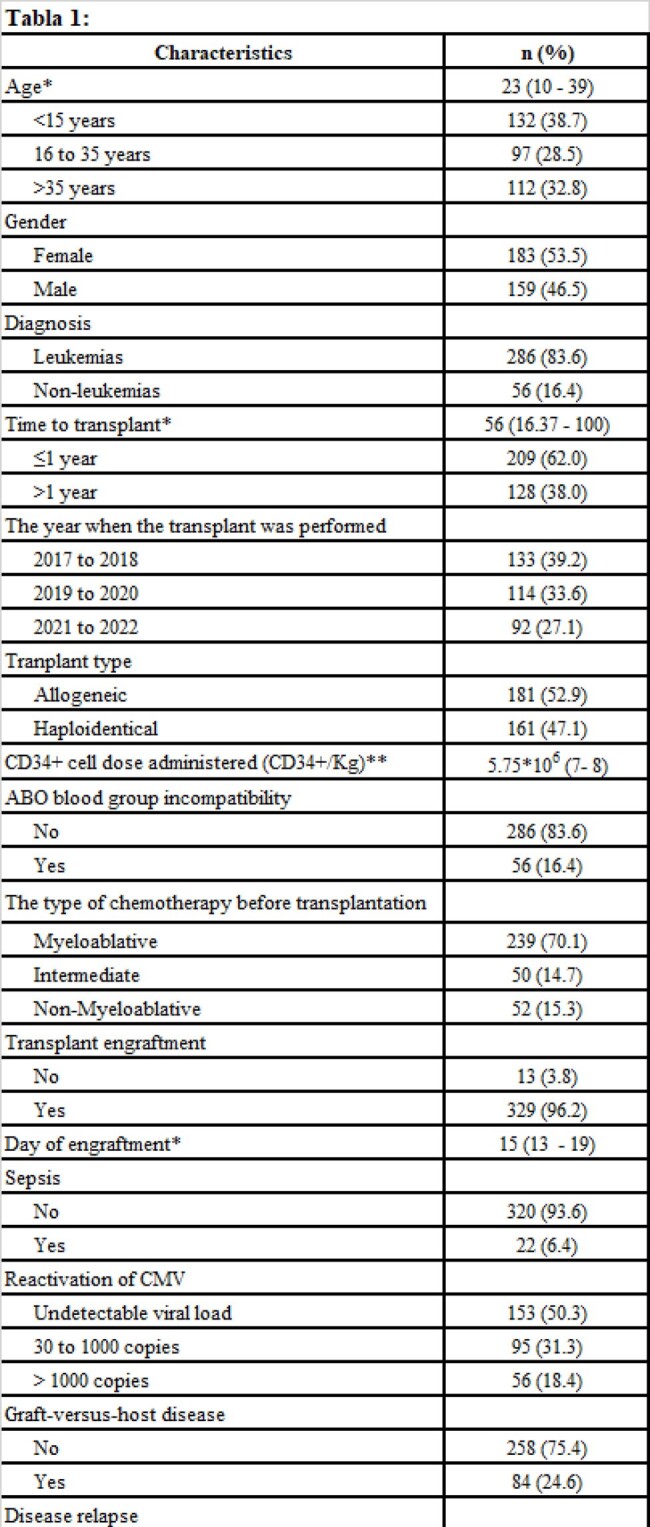

**Methods:**

Retrospective cohort study, conducted at a hospital in Peru between April and May 2024. We included all patients who underwent hematopoietic stem cell transplant between 2017 and 2022. We evaluated death at one hundred days of follow-up and at two years.

Survival at 100 days post transplant

**Figure:**
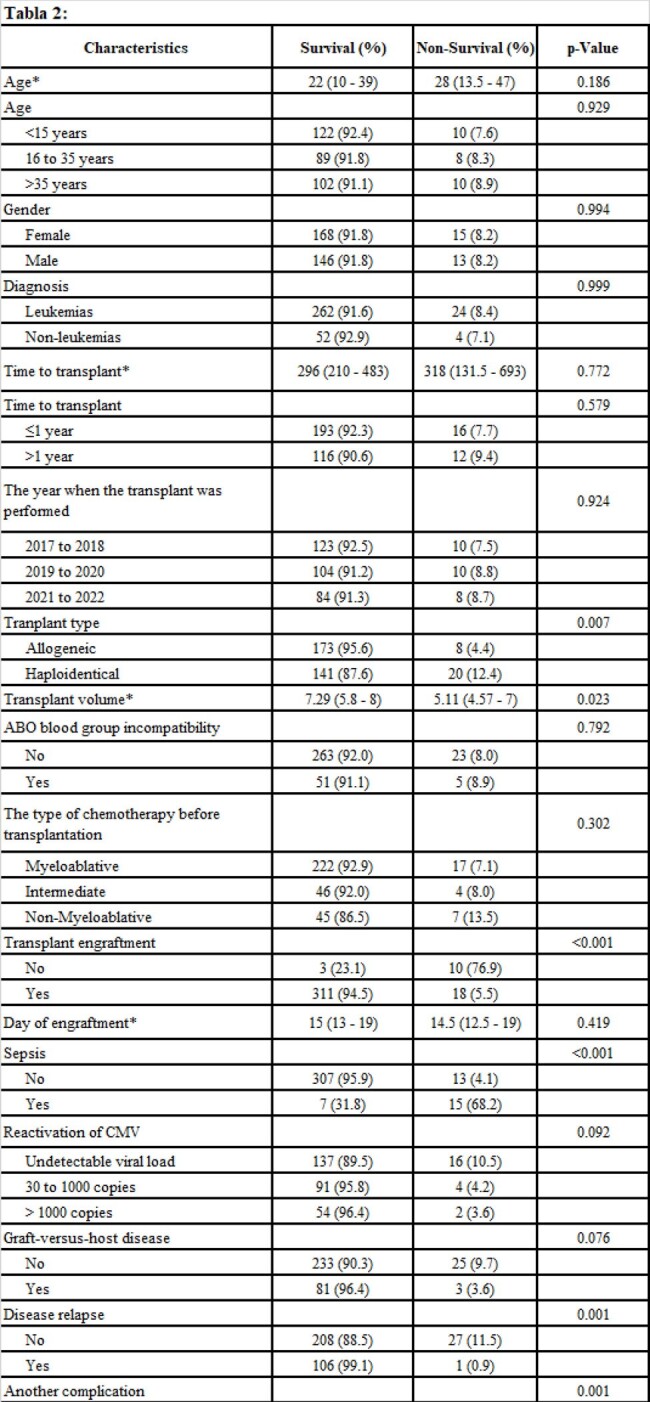

**Results:**

We evaluated 342 medical records, median age 23 years, 53.5% were women. The most common diagnosis for the transplant was leukemia 83.6%. Mortality in the first 100 days post TPH was 8.2%, and at two years was 30.4%. In the multivariate model and at one hundred days, sepsis HRa: 24.72 (7.95 to 76.85) was associated with death and engraftment to survival HRa: 0.26 (0.07 to 0.93). Regarding mortality at two years, haploidentical transplant HRa:1.92 (1.20 to 3.08), sepsis HRa: 5.36 (2.98 to 9.63), and disease relapse HRa: 4.32 (2.80 to 6.66) were associated with death and engraftment to survival HRa: 0.12 (0.06 to 0.25).

2-year post-transplant survival

**Figure:**
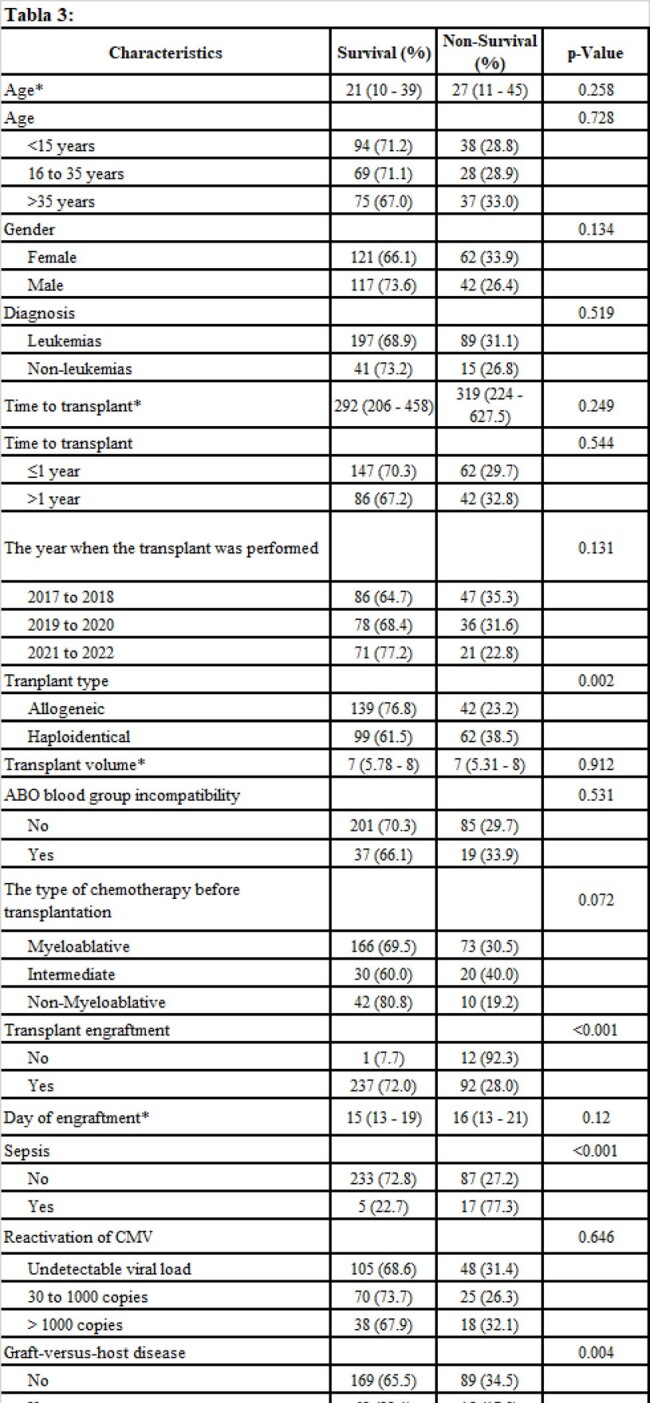

**Conclusion:**

Sepsis was the primary risk factor associated with death in the first one hundred days post-transplant. At two years of follow-up, disease relapse and sepsis were the risk factors for death. Hematopoietic stem cell transplant in Peru is performed in few specialized centers and this initial report identifies the need for further interventions to reduce the risk of infection.

**Figure:**
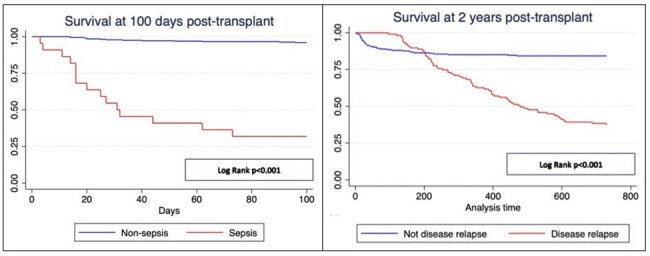

**Disclosures:**

All Authors: No reported disclosures

